# Expression, Functional Polymorphism, and Diagnostic Values of MIAT rs2331291 and H19 rs217727 Long Non-Coding RNAs in Cerebral Ischemic Stroke Egyptian Patients

**DOI:** 10.3390/ijms25020842

**Published:** 2024-01-10

**Authors:** Tarek K. Motawi, Nermin Abdel Hamid Sadik, Olfat G. Shaker, Maggy Maged Haider Ghaleb, Eman M. Elbaz

**Affiliations:** 1 Department of Biochemistry, Faculty of Pharmacy, Cairo University, Cairo 11562, Egypt; 2 Medical Biochemistry and Molecular Biology Department, Faculty of Medicine, Cairo University, Cairo 11562, Egypt; 3 Faculty of Pharmacy, Cairo University, Cairo 11562, Egypt

**Keywords:** cerebral ischemic stroke, LncRNA, MIAT, H19, epigenetic methylation

## Abstract

Cerebral ischemic stroke (CIS) is a severe cerebral vascular event. This research aimed to evaluate the role of single-nucleotide polymorphisms (SNPs) of the lncRNAs MIAT rs2331291 and H19 rs217727 and epigenetic methylation in the expression patterns of serum lncRNA H19 in CIS Egyptian patients. It included 80 CIS cases and 40 healthy subjects. Serum MIAT expression levels decreased, whereas serum H19 expression levels increased among CIS compared to controls. For MIAT rs2331291, there were significant differences in the genotypic and allelic frequencies between the CIS and healthy subjects at *p* = 0.02 and *p* = 0.0001, respectively. Our findings illustrated a significantly increased MIAT T/T genotype frequency in hypertensive CIS compared to non-hypertensive CIS at *p* = 0.004. However, H19 rs217727 gene frequency C/C was not significantly higher in non-hypertensive CIS than in hypertensive CIS. The methylation of the H19 gene promoter was significantly higher in CIS patients compared to healthy subjects. The level of MIAT was positively correlated with serum H19 in CIS. Receiver operating characteristics (ROC) analysis revealed that serum MIAT and H19 have a high diagnostic potential for distinguishing CIS subjects from healthy ones. In conclusion, the MIAT-rs2331291 polymorphism might serve as a novel potential indicator of CIS.

## 1. Introduction

Stroke stands as the primary reason for morbidity and mortality worldwide [1]. It affects all age groups, with occurrence rates rising by age [2,3]. Its lifetime risk is estimated at one in four by the age of 80 [4]. Additionally, stroke is ranked third among the causes of death in Western states [5,6]. In Egyptian society, the crude prevalence rate of stroke is very elevated, with a rate of 963 per 100,000 individuals [7]. The WHO reports that stroke affects 15 million patients across the world and causes permanent disability for nearly 5 million [8]. Broadly, there are two types of strokes: ischemic (85%) and hemorrhagic (15%). Acute stroke and acute coronary syndromes have many similarities [9,10].

Cerebral ischemic stroke (CIS) happens in the case of interrupting cerebral blood flow (CBF) because of embolism or thrombosis. The sudden interruption of CBF results in cell necrosis, and cerebral edema follows, destroying the blood–brain barrier (BBB) [11,12,13]. Releasing necrotic cells prompts inflammatory cytokines and apoptosis, resulting in the cell death of half cells in the infarction, aggravating brain injury [14,15].

Various complex factors help destroy the BBB after CIS and aggravated brain injury, such as oxidative stress, excitatory amino acids’ toxicity, excessive calcium ions, augmented apoptosis, and inflammation [12,16,17,18,19]. However, the absence of rapid prognostic and diagnostic tests seems to be an essential drawback for decision-making regarding managing strokes. Diagnosing CIS early can be facilitated by elucidating genetic and epigenetic factors and biomarkers [20].

Imaging techniques and clinical examinations are essential for diagnosing strokes [21]. Small infarcts are hard to report within six hours of onset, despite the sensitivity and specificity of imaging-based techniques [18,22]. Additionally, these diagnosis techniques are expensive and problematic, especially for cases from faraway and poor regions. Early assessment, which may help estimate the prognosis and severity of CIS, is critical to elevating care to improve the outcome. Consequently, the development of a non-invasive, highly sensitive blood biomarker for the early monitoring and screening of brain ischemia cases is significant.

Long non-coding RNAs (lncRNAs) are described as RNA molecules (>200 nucleotides) in length that do not encode proteins [23,24]. Over 50,000 human lncRNAs have been identified since the 1990s [25,26]. LncRNAs are significantly used in initial biological processes, including growth, development, reproductive health, and tissue regeneration [27,28]. Nonetheless, the functions and potential mechanisms of lncRNAs are not completely disclosed in the complex pathophysiological processes of CIS [28,29]. LncRNAs are vital for regulating the expression level of protein-coding genes and relevant signaling pathways in developing several illnesses at many levels, e.g., transcriptional regulation, epigenetic regulation, and post-translational control [30,31].

LncRNAs can help regulate the pathophysiologic processes of CIS due to their altered expressions revealed in blood samples of severe CIS cases, animal models of focal cerebral ischemia, and oxygen-glucose deprivation (OGD) cell models [28]. Due to their potentially significant role, lncRNAs can serve as valuable biomarkers for the purposes of diagnosing, treating, and prognosticating CIS.

The ability of lncRNAs to interact with DNA and RNA immediately and with little difficulty is attributed to their distinctive structural features [32]. They can engage in interactions with several proteins through the formation of complicated secondary structures [33,34]. Based on the subcellular localizations of lncRNAs, they have been observed to exhibit expression within the nuclear or cytoplasm [35]. In the nucleus of the cell, LncRNAs can make use of chromatin remodeling complexes at specific locations on the chromosome to prompt epigenetic gene silencing [36,37]. Consequently, they can control the expression of particular genes that are situated either on the same chromosome or on a different one [38]. Cytoplasmic lncRNAs serve as a molecular sponge against microRNAs to influence microRNAs’ expression and function [39,40] or target mRNAs indirectly or directly to regulate transcriptional activity [41].

Single-nucleotide polymorphisms (SNPs) are important for identifying the risk of a person’s vulnerability to different diseases and responses to drugs [42]. The current endeavor of research involves the identification of commonly occurring SNPs that are biologically significant, particularly those that exhibit associations with the risks of disease. The necessity of identifying and characterizing a substantial quantity of these SNPs is imperative before their widespread utilization as genetic tools by researchers [43]. Due to the considerable variation in SNP allele frequencies among human ethnic communities and groups, the utilization of an ethnically varied panel has been chosen as a means to enhance the likelihood of SNP discovery [43,44].

The key expression of myocardial infarction–associated transcript (MIAT) often occurs in heart and fetal brain tissues [45]. It is a particularly intriguing functional factor in all lncRNAs associated with diseases and shows deregulation in several illnesses, such as upregulation in myocardial infarction [46,47], diabetic cardiomyopathy [48,49], lung cancer [50,51], and CIS [52]. MIAT was reported to be a key controller of vascular integrity and neuronal function [52,53]. The development of CIS is significantly influenced by neurovascular dysfunction [54]. Nevertheless, the role of MIAT in CIS has not been defined yet. Ishii et al. [55] found that altered expression of lncRNA MIAT due to an SNP in MIAT rs2331291 is significantly associated with the development of myocardial infarction. However, the role of polymorphisms in MIAT in CIS patients is still unclear.

LncRNA-H19 is widely recognized as an extensively studied and well-defined lncRNA gene. It is highly expressed in embryogenesis and subsequently declines after birth, but can be reactivated in response to hypoxic conditions [56,57]. Therefore, it might participate in the pathogenesis of CIS. It was found that circulating H19 levels were significantly higher among CIS cases compared to healthy controls [58].

SNPs of the H19 gene were found to modify its expression and are related to risk factors for cardiovascular and cerebrovascular diseases, e.g., obesity and hypertension [4,59,60]. Various levels were reported for H19, rs217727, to be related to the higher risk of CIS [61]. Moreover, the hypermethylation in the promoter of the H19 gene and allele-specific methylation of the 3′ portion of H19 is possibly associated with the changed expression of H19 [62,63].

Therefore, this study aims to evaluate the role of SNPs of the lncRNAs H19 rs217727 and MIAT rs2331291 and epigenetic methylation in the expression patterns of serum lncRNA H19 levels in Egyptian patients with CIS.

## 2. Results

### 2.1. Demographic and Clinical Features of CIS Cases and Healthy Controls

The demographic profile and clinicopathological data of the 80 CIS cases (male = 62; female = 18) and 40 healthy control subjects (male = 31; female = 9) who participated in the research are shown in Table 1. Table 2 shows the demographic profile and clinicopathological data of the CIS patient subdivisions.

According to Table 3, the genotype distribution of MIAT-rs2331291 and H19 rs217727 polymorphisms did not agree with those expected for the Hardy–Weinberg equilibrium at *p* = 0.005 and *p* = 0.048, correspondingly, among CIS cases.

However, the genotype distribution of the MIAT-rs2331291 and H19 rs217727 polymorphisms matched those expected for the Hardy-Weinberg equilibrium at *p* = 0.12 and *p* = 0.098, correspondingly, among hypertensive CIS cases and at *p* = 0.098 and *p* = 0.273, correspondingly, in non-hypertensive CIS cases (Table 4).

Regarding the subgroup, among diabetic hypertensive CIS cases, the genotype distribution of MIAT-rs2331291 polymorphisms does not match the expectations for the Hardy–Weinberg equilibrium at *p* = 0.026. Nevertheless, the genotype distribution of H19 rs217727 polymorphisms among cases of diabetic hypertensive CIS matches the Hardy–Weinberg equilibrium at *p* =0.094. Furthermore, among non-diabetic hypertensive CIS cases, the genotype distribution of MIAT-rs2331291 and H19 rs217727 polymorphisms, respectively, matches the Hardy–Weinberg equilibrium at *p* = 0.964 and *p* = 0.548 (Supplementary Table S1).

In the diabetic non-hypertensive CIS group, the distribution of MIAT-rs2331291 and H19 rs217727 polymorphisms does not significantly deviate from the Hardy–Weinberg equilibrium (*p* = 0.271 and *p* = 0.396, respectively). In the non-diabetic non-hypertensive CIS group, the distribution of the -rs2331291 and H19 rs217727 polymorphisms does not significantly deviate from the Hardy–Weinberg equilibrium (*p* = 0.285 and *p* = 0.48, respectively) (Supplementary Table S2).

Regarding MIATrs2331291, the genotypic and allelic frequencies demonstrated significant differences between the CIS cases (*p* = 0.02) and the healthy ones (*p* = 0.0001). Concerning H19-rs217727, the genotypic and allelic frequencies did not demonstrate significant differences between the CIS cases and the healthy control subjects at *p* = 0.22 and *p* = 0.25, correspondingly (Table 5).

Among hypertensive CIS cases, the T/T genotype of the MIAT- rs2331291 gene was observed in 27.5% (11/40) individuals, but C/T and C/C were reported in 37.5% (15/40) and 35% (14/40) individuals, respectively. In non-hypertensive CIS cases, T/T, C/T, and C/C were observed in 7.5% (3/40), 22.5% (9/40), and 70% (28/40) of individuals, respectively. A significantly higher MIAT T/T genotype frequency (11/40, 27.5%) was found in hypertensive CIS patients than in non-hypertensive CIS patients (3/40, 7.5%) at *p* = 0.004. Moreover, there was a significant difference in the allele frequencies (*p* = 0.0001) between hypertensive and non-hypertensive CIS cases (Table 6).

Regarding H19 rs217727 gene polymorphism, the C/C genotype was observed in 70% (28/40) hypertensive CIS cases, while T/C and T/T were found in 22.5% (9/40) and 7.5% (3/40) of participants, respectively. In non-hypertensive CIS cases, C/C, T/C, and T/T were observed in 72.5% (29/40), 22.5% (9/40), and 5% (2/40) of participants, respectively. The H19 rs217727 gene frequency C/C was higher in non-hypertensive CIS patients (29/40, 72.5%) than in hypertensive CIS patients (28/40, 70%) at *p* = 0.89. No significant difference in the allele frequencies (*p* = 0.677) was observed between hypertensive and non-hypertensive CIS cases (Table 6).

Concerning the subgroup MIAT rs2331291, neither the genotypic nor allelic frequencies (*p* = 0.25, *p* = 0.823, and *p* = 0.511, *p* = 0.195, respectively) between the diabetic and non-diabetic hypertensive CIS cases nor between the diabetic and non-diabetic non-hypertensive CIS cases demonstrate any statistically significant differences (Supplementary Table S3).

Regarding H19 rs217727, there are no statistically significant differences between the genotypic and allelic frequencies of the diabetic and non-diabetic hypertensive CIS cases (*p* = 0.8, and *p* = 0.775, respectively) or the diabetic and non-diabetic non-hypertensive CIS cases (*p* = 0.989, and *p* = 0.927, respectively) (Supplementary Table S3).

### 2.2. Serum Expression Levels of lncRNA-MIAT and H19 Levels in CIS Patients and Healthy Subjects

The serum expression level of lncRNA-MIAT level was significantly lower (0.569 ± 0.94, mean ± SD) in CIS cases than in the healthy subjects at *p* = 0.0001. Nonetheless, the serum expression level of lncRNA-H19 levels in CIS patients (23.19 ± 20.87, mean ± SD) was significantly higher than in healthy controls at *p* = 0.0001 (Table 7).

Furthermore, a non-significant decrease in the serum lncRNA-MIAT level was found in hypertensive CIS patients compared to non-hypertensive CIS patients (*p* = 0.08). Interestingly, serum lncRNA-H19 levels significantly declined in hypertensive CIS patients (*p* = 0.002) compared to non-hypertensive CIS patients (Table 7).

In the MIAT gene, the serum level of the MIAT rs2331291 CC genotype (n = 42) was non-significantly increased among CIS cases compared to healthy controls (0.77 ± 1.2, mean ± SD). In the H19 gene, the serum H19 level rs217727 CT genotype (n =18) was non-significantly increased among CIS cases in comparison with healthy subjects (28.3 ± 24.9, mean ± SD), as shown in Table 8.

Regarding the rs2331291 TT genotype group (n = 11), Table 9 demonstrates that the serum MIAT level was not significantly higher in hypertensive CIS patients compared to non-hypertensive CIS patients (0.61 ± 0.51; vs. 0.39 ± 0.17; mean ± SD). However, there was no statistically significant difference between the hypertensive CIS patients and non-hypertensive CIS patients in the serum H19 level for the rs217727 CC genotype group (n = 28) (15.33 ± 11.8; vs. 27 ± 22.8; mean ± SD)

### 2.3. Different Methylation Patterns of H19 Gene Promoter in CIS Patients

The methylation states of lncRNA-H19 in the groups are shown in Table 10. The methylation rate showed a significant increase in CIS cases compared with healthy subjects (*p* = 0.0001). Nevertheless, no significant differences were reported between allele frequencies between CIS subjects (*p* = 0.817) and healthy subjects (*p* = 0.261) or hypertensive and non-hypertensive CIS patients (*p* = 0.598, *p* = 0.854, respectively).

### 2.4. Correlations between Serum lncRNA-MIAT and H19 Levels

**Figure 1:**
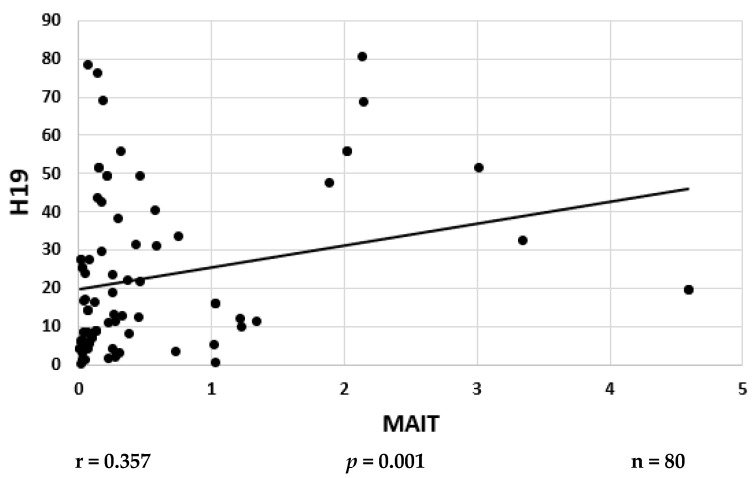
Correlation analysis between serum lncRNA MAIT and lncRNAH19 levels in CIS patients. Correlation was determined using Spearman correlation, r = Spearman rho coefficient.

An observed positive correlation (r = 0.357) was recorded between the serum levels of MIAT and H19 in CIS cases at *p* = 0.001 (Figure 1). However, the serum levels of MIAT and H19 did not significantly correlate in the hypertensive CIS cases (n = 40, r = 0.284, *p* = 0.07). Moreover, the serum levels of MIAT and H19 in the non-hypertensive CIS patients did not significantly correlate (n = 40, r = 0.259, *p* = 0.107).

The serum levels of MIAT and H19 significantly correlated in the subdivision groups for non-diabetic hypertensive CIS cases (r = 0.646, *p* = 0.002) but not for diabetic hypertensive CIS cases (r = 0.045, *p* = 0.85). However, neither the diabetic (r =0.17, *p* = 0.945) nor the non-diabetic non-hypertensive (r = 0.422, *p* = 0.064) CIS cases showed a statistically significant correlation between the serum levels of MIAT and H19 (Table 11).

### 2.5. ROC Curve Analysis

**Figure 2:**
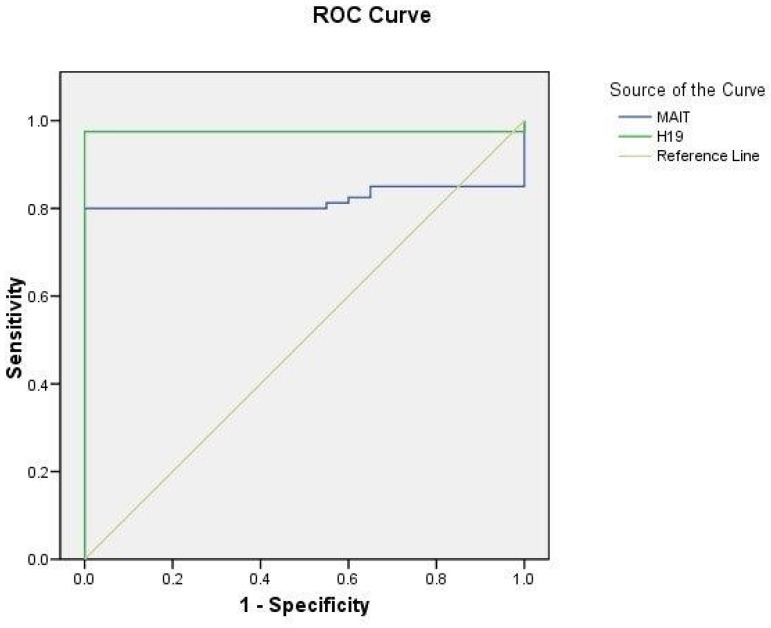
ROC curve analysis of serum lncRNA MIAT and H19 levels in CIS patients (n = 80) and healthy controls (n = 40).

The ROC curve helped to determine the significance of serum MIAT and H19 levels as probable diagnostic biomarkers for CIS cases (n = 80) and control subjects (n = 40). The serum MIAT level at the cutoff value of 1.21 had a sensitivity of 80% and a specificity of 100% with an area under the curve (AUC) of 0.819 (95% CI = 0.73–0.9) at *p* < 0.0001. Additionally, the serum H19 level in the cutoff value of 1.24 had a sensitivity and specificity of 97.5% and 100%, correspondingly with an AUC of 0.975 (95% CI = 0.94–1.009) at *p* < 0.0001 (Figure 2).

**Figure 3:**
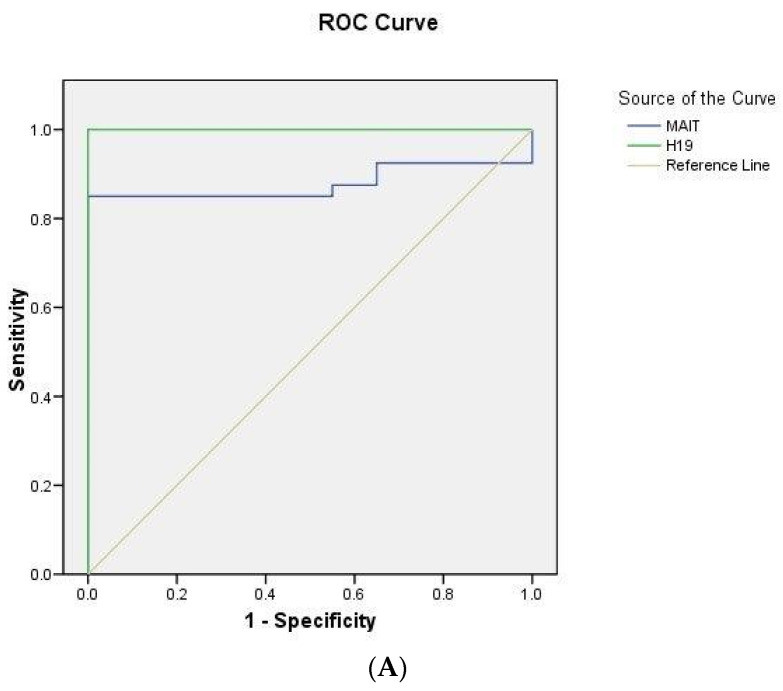

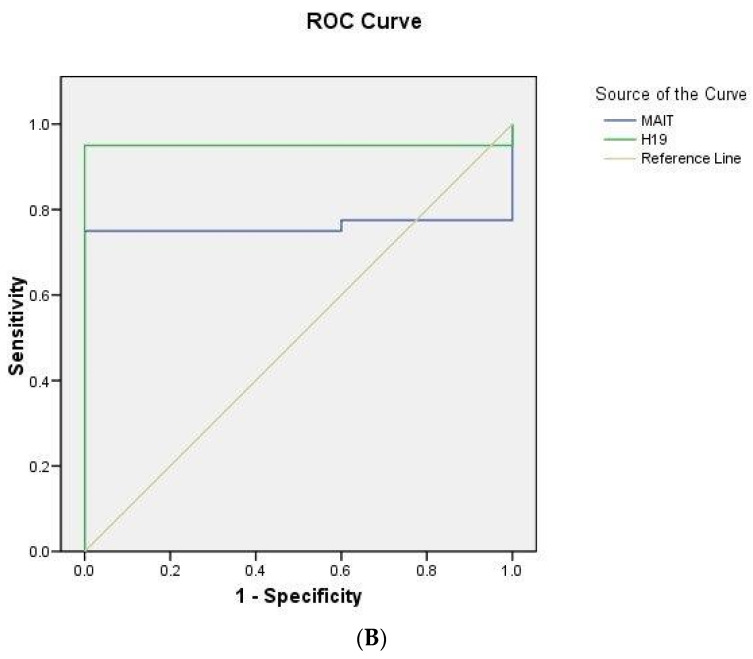
(**A**) ROC curve analysis of serum lncRNA MIAT and H19 levels in hypertensive CIS patients (n = 40) and healthy controls (n = 40). (**B**) ROC curve analysis of serum lncRNA MIAT and H19 levels in non-hypertensive CIS patients (n = 40) and healthy controls (n = 40).

Moreover, the study demonstrated that serum MIAT characterized hypertensive CIS cases from healthy control subjects with an AUC of 0.879 (95% CI = 0.87–0.97) at *p* < 0.0001, and the optimal sensitivity and specificity were reported to be 85% and 100%, respectively, at a cutoff value of 1.21. Serum H19 could be a potential biomarker for distinguishing hypertensive CIS cases from healthy control subjects with an AUC of 1 (95% CI = 1–1) at *p* < 0.0001, conferring 100% sensitivity and 100% specificity at a cutoff value of 1.24 (Figure 3A).

For the diagnostic values of serum MIAT between non-hypertensive CIS patients and healthy controls, ROC curve analysis was performed with an AUC of 0.76 (95% CI = 0.63–0.89) at *p* < 0.0001 and optimal sensitivity and specificity of 75 and 100%, respectively, with a cutoff value of 1.39. As for the diagnostic values of serum H19 between non-hypertensive CIS cases and healthy control subjects, the AUC was 0.95 (95% CI = 0.88–1.01) at *p* < 0.0001 and the optimal sensitivity and specificity were 95 and 100%, respectively, with a cutoff value of 1.249 (Figure 3B).

**Figure 4:**
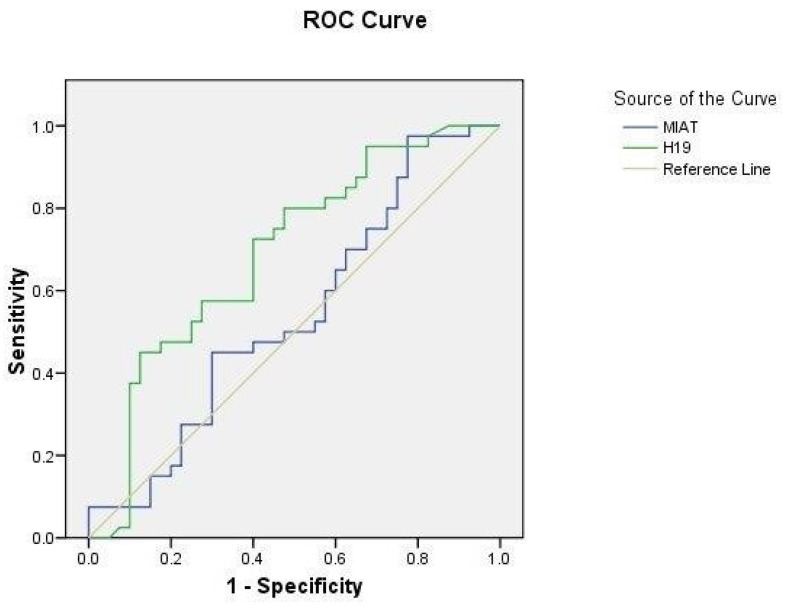
ROC curve analysis of serum lncRNA MIAT and H19 levels in hypertensive CIS patients (n = 40) and non-hypertensive CIS patients (n = 40).

To evaluate the ability of serum lncRNA MIAT and H19 levels to differentiate between hypertensive and non-hypertensive CIS cases, ROC curve analysis revealed an AUC of 0.542 (95% CI = 0.414–0.67) at *p* = 0.519 and 0.69 (95% CI = 0.572–0.8) at *p* = 0.003, respectively (Figure 4).

## 3. Discussion

Stroke mainly causes mortality and lasting disability in developed states [64]. Therefore, developing a non-invasive, highly sensitive blood biomarker for the early monitoring and screening of cerebral ischemia patients is significant. LncRNAs can control gene expression at various levels, including transcriptional, epigenetic, and post-transcriptional regulation [65]. Notably, previous studies have suggested that SNPs are linked to the development of stroke [66]. SNPs typically exist all across a person’s DNA [67]. Approximately 4 to 5 million SNPs in an individual’s genome are reported, implying that they usually happen once per 1000 nucleotides [67]. They help predict the person’s reaction to some medications, susceptibility to environmental factors, e.g., toxins, as well as the risk of developing diseases, including CIS [43].

**Figure 5:**
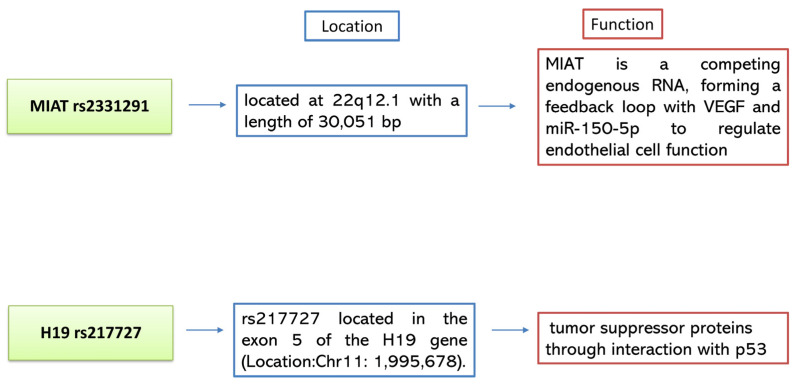
Summary of location and known functions of MIAT rs2331291 and H19 rs217727 long non-coding RNAs.

The MIAT gene is located at 22q12.1 with a length of 30,051 bp (Figure 5) [65]. Unlike other nuclear-retained lncRNA, MIAT exhibits a lack of a relationship with chromatin and closely relates to the nuclear matrix [68]. Functional studies have previously reported reduced MIAT expression levels inhibiting cell proliferation in breast cancer cells [69] and malignant B cells [70]. MIAT serves as a competing endogenous RNA (ceRNA) and creates a feedback loop for miR-150-5p to control lymphatic endothelial cell functions, including apoptosis [53,71,72]. According to Yan et al. [71], MIAT could bind miR150-5p in endothelial cells and inhibit the degradation of their direct target vascular endothelial growth factor (VEGF). Their results indicated that specific variants in the MIAT lncRNA could adjust the structure of MIAT, increasing the binding of miR-150-5p and, in turn, hindering the degradation of the target genes, e.g., VEGF [71,72]. Jiang et al. [53] confirmed that MIAT knockdown caused cerebral microvascular degeneration, progressive neuronal loss, and neurodegeneration, as well as behavioral deficits in a CNS neurovascular disorder, Alzheimer’s disease. This observation demonstrated that MIAT downregulation is related to increased apoptosis levels among CIS cases.

During an ischemic stroke, the cessation of blood supply to a specific area of the brain results in the deprivation of oxygen and glucose to the adjacent tissue, which leads to a disruption in the synthesis of ATP and energy failure, causing the disturbance of ion homeostasis and acid–base balance [73,74]. Additionally, the suppression of oxidative phosphorylation triggers the elevated generation of free radicals along the mitochondrial respiratory chain, augments intracellular Na^+^, and eventually results in membrane depolarization after the depletion of ATP substrate for the Na^+^-K^+^ pump. The sudden and entire break of transmembrane ion gradients, as well as neuronal edema and mitochondrial disruption, mark the spread of depolarizations, playing a role in excitotoxicity and neuronal cell death [75,76]. Such waves of sustained depolarization, called cortical spreading depression, have correlations with the synaptic release of glutamate, a key excitatory neurotransmitter, and its electrogenic transport from depolarized astrocytes. The substantial rise in extracellular glutamate causes the overstimulation of many receptors, including α-amino-3-hydroxy-5-methyl-4-isoxazole propionic acid (AMPA), kainate, members of the acid-sensing ion channel (ASIC), metabotropic, and NMDA-type glutamate receptors. Therefore, the influx of Ca^2+^ and Na^+^ ions via the channels fenced by those receptors takes place [77]. Ultimately, the increasing intracellular Ca2+ induces activating secondary signal drops, including many proteases, lipases, and kinases, leading to organelle dysfunction and many cell death pathways, e.g., apoptosis [78,79].

Genome-wide association studies (GWASs) illustrated that MIAT rs2331291 in intron 15,338 is related to altered vulnerability to myocardial infarction [55,80]. Our study found that the MIAT rs2331291 genotype and allele frequencies differed significantly between CIS cases and healthy ones. To the best of our knowledge, MIAT rs2331291 has not previously been measured in CIS patients. Our results revealed that the MIAT rs2331291 T allelic frequency showed a significant increase in CIS cases compared to healthy controls (32.5% vs. 8.8%) and could be a risk factor for CIS compared with the C allelic frequency.

The LncRNA-H19 gene affected cell death, apoptosis, and angiogenesis during CIS [81]. We observed a significant increase in the lncRNA-H19 serum expression level between CIS cases and healthy ones. This observation agrees with Wang et al. [82,83]. The increase in H19 levels was reported to inhibit dual specificity phosphatase 5 (DUSP5), thus activating extracellular signal-regulated kinase 1/2 (ERK1/2) and autophagy, causing autophagic death of neurons [84,85]. Moreover, this increase might be attributed to neuroinflammation, as lncRNA-H19 is a significant controller in the progression of atherosclerosis, which can lead to CIS as it causes neuroinflammation by influencing histone deacetylase 1-dependent M1 microglial polarization [82]. In contrast, acid phosphatase 5 (ACP5) was found to be a direct target gene of H19, suggesting the positive regulatory effect of H19 on ACP5 expression, thus promoting lipid synthesis and causing CIS [86,87]. GWAS revealed that H19 rs217727, located at 11p15.5, was associated with altered susceptibility to systolic blood pressure and coronary artery disease [80]. The different values of H19 rs217727 were related to the higher risk of ischemic stroke [83]. However, our results revealed that there were no significant differences in the H19 rs217727 genotype and allele frequencies between CIS cases and healthy ones, and this finding matched that of Zhu et al. [88].

In general, risk factors and the gene expression of CIS are regulated by methylation alterations in genes through many pathological processes, including disturbances in the coagulation cascade, elevated plasma homocysteine levels, dyslipidemia, atherosclerosis, and inflammatory responses [89]. As an illustration, the regulation of the essential lipid profile enzyme is governed by apolipoprotein E (APOE) [90]. It was observed that the APOE genotype, particularly the E4 allele, exhibited a correlation with elevated levels of LDL-C and carotid intima-media thickness [91]. The abnormal expression of the APOE gene and subsequent dyslipidemia, which could result in an earlier onset of stroke, could be attributed to the hypermethylation of the APOE promoter [92,93]. Furthermore, DNA methylation could increase the potential concentration of plasma homocysteine, a well-known risk factor for strokes [94].

The results illustrated that the methylation rate of the H19 gene significantly increased among CIS cases compared with healthy controls. However, we failed to find any significant difference between H19 rs217727 gene allele frequencies in hypertensive and non-hypertensive CIS patients. It is worth noting that the expression of H19 inhibits S-adenosylhomocysteine hydrolase (SAHH), which hydrolyzes S-adenosylhomocysteine (SAH), which, sequentially, acted as an inhibitor of S-adenosylmethionine (SAM)-dependent methyltransferases, decreased DNA methylation potential caused by hyperhomocysteinemia, and decreased the SAM:SAH ratio [95]. Devlin et al. [96] observed that a lower brain and hepatic SAM:SAH ratio was paradoxically associated with higher H19 DNA methylation, which corroborated our results for CIS patients. On the other hand, Jiang Y et al. [97] noted a higher aortic SAM:SAH ratio with a decrease in DNA methylation, indicating the tissue-specific influence of hyperhomocysteinemia on H19 methylation.

## 4. Subjects and Methods

### 4.1. Subjects

Eighty subjects were recruited for this case-control study successively from the Stroke Unit, Neurology Department, Kasr El-Aini Hospital, from January 2021 to January 2022. CIS diagnosis relied on clinical manifestations and was supported by computed tomography (CT) and magnetic resonance imaging (MRI) brain scans. They were equally subdivided into hypertensive and non-hypertensive CIS patients. Each subdivision was further divided into diabetic and non-diabetic groups. Exclusion criteria encompassed patients who exhibited established causes for ischemic stroke, such as systemic malignancy, systemic lupus erythematosus, coronary artery disease, circulatory problems such as heart failure, and kidney illness such as chronic kidney disease, as well as cases with a history of hemorrhagic stroke, traumatic brain injury, brain tumor, or other conditions characterized by brain inflammation. The control group of 40 volunteers seemed healthy with no history of stroke or other neurological conditions that matched the patient subject group in the distribution of sex and age.

### 4.2. Blood Sampling and Laboratory Assays

Trained laboratory personnel took venous blood samples of approximately 10 mL in volume. A blood portion was left to clot and then centrifuged at 3500 rpm for five minutes to separate the serum, utilized to evaluate the total cholesterol, triglyceride (TG), low-density lipoprotein (LDL), and high-density lipoprotein (HDL). The assays were carried out using a Roche Hitachi Chemistry Analyzer (. Additionally, another portion of the blood sample was collected in vacutainer tubes with ethylenediaminetetraacetic acid (EDTA) and stored at −80 °C for the molecular assays.

### 4.3. DNA Extraction and Genotyping

The QIAamp DNA Blood MiniKit (Qiagen, Hilden, Germany) was utilized to extract genomic DNA from the entire EDTA blood samples of all participants, in accordance with the guidelines of the manufacturer. The DNA yield and purity were assessed by NanoDrop 2000 (ThermoFisher Scientific, Foster City, CA, USA). Moreover, a TaqMan allelic discrimination assay was employed for SNP genotyping by real-time PCR, aided by predesigned primer/probe sets for SNPs: rs2331291 T/C [assay ID: C-2467709-10, Lot: P180720-003-F07] at MIAT, while rs217727 C/T [assay ID: C-2603707-10, Lot: P180720-003-F08] at H19 was used (ThermoFisher Scientific, Foster City, CA, USA) [98]. The reaction was conducted in (25 μL) 12.5 μL of TaqMan master mix, 1.25 μL of primer/probe, 1 μL of DNA, and 10.25 μL of H_2_O. In addition, amplifications were carried out on the Rotor-Gene Q System (Qiagen, Hilden, Germany) at 95 °C for 10 min, followed by 45 cycles at 92 °C for 15 s and 60 °C for 90 s.

### 4.4. Serum MIAT and H19 Assays by RT-qPCR

The extraction of total RNA from the serum of all subjects was performed using an miRNeasy extraction kit (Qiagen, Hilden, Germany). The purity and concentration of the extracted RNA were assessed using a NanoDrop 2000 (Thermo Fischer Scientific, Foster City, CA, USA). Then, the extracted RNA was utilized for lncRNA expression analysis. Regarding every single MIAT and H19, RT was performed with 0.1 μg of RNA in a 20 μL reaction volume by a high-capacity cDNA reverse transcription kit (Applied Biosystems, Foster City, CA, USA) following the manufacturer’s guidelines. The Maxima SYBR green qPCR master mix (Thermo Fischer Scientific, Foster City, CA, USA) was utilized to measure the expression of MIAT and H19, and the values of RT-PCR products were standardized concerning GAPDH (internal control). The primer sequences utilized in this study were as follows [33,43]:

MIAT: forward 5′-ATCACGCGTCCAGAGTCAGGGAAAAAGACC-3′
MIAT: reverse 5′-ATCCTCGAGTTGAATTCTACCATTTTCT TACATC-3′
H19: forward 5′-GTCGCTATCTCTAGGTGAAG-3′
H19: reverse 5′-GTGGAGGCTTTGAATCTCTC-3′
GAPDH: forward 5′-CCCTTCATTGACCTCAACTA-3′
GAPDH: reverse 5′-TGGAAGATGGTGATGGGATT-3′

A reaction mixture of a 20 μL final volume was utilized for RT-PCR, as previously illustrated [99]. RT-PCR was carried out using a rotor gene Q system: initial denaturation at 95 °C for 10 min and then 40 PCR cycles of 95 °C for 15 s and 60 °C for 60 s. Gene expression related to the internal control (2^−Δ(Ct)^) was determined. After that, the fold change was determined by 2^−ΔΔ(Ct)^ [100].

### 4.5. H19-rs217727 Promoter Methylation

H19-rs217727 promoter methylation was performed using the Global DNA Methylation Assay Kit (5 Methyl Cytosine, Colorimetric) (ab233486, Abcam, Cambridge, UK) as follows: 100 µL of binding solution was added to 2 µL of negative control and 2 µL of sample DNA, respectively. The plate was gently shaken, covered, and incubated at 37 °C for 60 min. The binding solution was removed from each well after 60 min of incubation, and each well was washed with 150 µL of the diluted 1X wash buffer three times. Then, 50 µL of the 5-mC detection complex solution was added to each well, covered, and incubated at room temperature for 50 min. After that, the 5-mC detection complex solution was removed from each well, and each well was washed with 150 µL of the diluted 1X wash buffer five times. Finally, 100 µL of developer solution was added to each well. The plate was gently shaken and incubated at room temperature. After 5 min, the developer solution turned blue in the presence of sufficient methylated DNA. The color in the negative control wells remained unchanged. Next, 100 µL of the stop solution was added to each well and mixed in order to stop the enzyme reaction. After 2 min of adding the stop solution, the color changed to yellow, and the absorbance was read on a microplate reader at 450 nm within 15 min. The percentage of methylated DNA was calculated using the following formula:

5 − mC%= (Sample OD − Negative Control OD/Slope × S) × 100%

S is the amount of input sample DNA in ng.

### 4.6. Statistical Analysis

SPSS 15 (IBM, Chicago, IL, USA) and GraphPad Prism 8.0 (GraphPad Software Inc., San Diego, CA, USA) were adopted to analyze data, which were statistically depicted as the mean ± standard deviation (SD), frequencies (number of cases), and percentages when applicable. The Kolmogorov–Smirnov test helped to test the numerical data for the normal assumption. Moreover, a comparison of numerical variables between the study groups was carried out by the Student *t*-test for independent samples. For the comparison of categorical data, a Chi-square (χ2) test was conducted. A Mann–Whitney U test or Kruskal–Wallis test and then Dunn’s multiple comparisons test were utilized to make a comparison of MIAT and H19 data because they were not normally distributed according to the Shapiro–Wilk and Kolmogorov−Smirnov normality tests. The Spearman coefficient was used to determine the correlations. Accuracy was represented by specificity and sensitivity. Receiver operator characteristic (ROC) analysis was utilized to determine the optimum cutoff value for the investigated diagnostic markers. Two-sided *p*-values of less than 0.05 were determined to be statistically significant. SNP Stats was used to carry out the analysis of SNP (Inistitut Català d’Oncologia, Barcelona, Spain; https://www.snpstats.net/start.htm, accessed on 18 November 2023).

## 5. Conclusions

In summary, the current findings confirm that H19 upregulation and MIAT downregulation levels are associated with CIS. In addition, the results revealed that the MIAT rs2331291 polymorphism might serve as a novel potential biomarker for CIS. Furthermore, the H19 rs217727 polymorphism could be significantly associated with CIS risk within the Egyptian population. Furthermore, the study found that the hypermethylation of the H19 promoter region might play a role in diagnosing CIS. However, we recommend conducting more research on SNPs to control the genes related to CIS susceptibility among CIS cases in Egypt.

## Figures and Tables

**Table 1:** Demographic profile and clinicopathological data of the CIS patients and healthy controls.

Parameter	CIS (n = 80)	Control (n = 40)	*p*-Value
**Sex**			
**Male**	62 (77.5%)	31 (77.5%)	0.99
**Female**	18 (22.5%)	9 (22.5%)	
**Age**	57.3 ±10.66	56.95 ± 10.71	0.73
**Total cholesterol**	211.5 ± 40.9	107 ± 23.3	0.001 *
**TG**	146.6 ± 61.3	90.9 ± 18.3	0.001 *
**LDL**	145.4 ± 40.9	73.3 ± 17.8	0.001 *
**HDL**	37.9 ± 11.9	54.4 ± 9.3	0.001 *
**Smoking**	51 (63.75%)	25 (62.5%)	0.89

Age is represented as mean ± SD, sex is represented as %. Abbreviations: cerebral ischemic stroke (CIS), triglyceride (TG), low-density lipoprotein (LDL), high-density lipoprotein (HDL). * Indicates a statistical significance.

**Table 2:** Demographic profile and clinicopathological data of the CIS patient subdivisions.

		Sex		*p*-Value	Age (Years)	*p*-Value	Smoking	Non-Smoking	*p*-Value
		Male (%)	Female (%)						
**CIS (n = 80)**	**HTN** **(n = 40)**	30 (75%)	10 (25%)	0.592	58.9 ± 8.88	0.162	25 (62.5%)	15 (37.5%)	0.816
	**Non- HTN** **(n = 40)**	32 (80%)	8 (20%)		55.6 ± 12		26 (65%)	14 (35%)	
**Hypertensive CIS** **(n = 40)**	**D.M (n = 20)**	13 (65%)	7 (35%)	0.27	63.3 ± 6.4	0.001 *	10 (50%)	10 (50%)	0.103
	**Non-D.M (n = 20)**	17 (85%)	3 (15%)		54.6 ± 8.9		15 (75%)	5 (25%)	
**Non- Hypertensive CIS (n = 40)**	**D.M (n = 18)**	14 (77.8%)	4 (22.2%)	1.0	60.2 ± 7.5	0.072	12 (67%)	6 (33%)	0.84
	**Non-D.M (n = 22)**	18 (81.8%)	4 (18.2%)		51.9 ± 13.9		14 (64%)	8 (36%)	

Data are expressed as mean ± SD or number (percentage). Abbreviations: Abbreviations: cerebral ischemic stroke (CIS), hypertensive (HTN), diabetic mellitus (D.M). * Indicates a statistical significance.

**Table 3:** Hardy–Weinberg equilibrium for MIAT-rs2331291 and H19-rs217727 in the CIS patients and healthy controls.

Genotype Allele	CIS			Control		
MIAT rs2331291	Observed Frequency	Expected Frequency	*p*-Value	Observed Frequency	Expected Frequency	*p*-Value
**CC**	52.5%	45.5%	0.005 *	82.5%	83.25%	0.54
**CT**	30%	43.9%		17.5%	16%	
**TT**	17.5%	10.56%		0.0%	0.75%	
**H19 rs217727**						
**CC**	71.3%	68%	0.048 *	57.5%	58.1%	0.8
**CT**	22.5%	28.8%		37.5%	36.2%	
**TT**	6.3%	3.12%		5%	5.7%	

Results are presented as percent. rs, reference single-nucleotide polymorphism (SNP) ID. Abbreviation: cerebral ischemic stroke (CIS), * Indicates a statistical significance at *p* < 0.05.

**Table 4:** Hardy-Weinberg equilibrium for MIAT-rs2331291 and H19-rs217727 in the hypertensive and non-hypertensive CIS patients.

Genotype Allele	HTN (n = 40)			Non-HTN (n = 40)		
MIAT rs2331291	Observed Frequency	Expected Frequency	*p*-Value	Observed Frequency	Expected Frequency	*p*-Value
**CC**	35%	28.93%	0.120	70%	66%	0.098
**CT**	37.5%	49.7%		22.5%	30.5%	
**TT**	27.5%	21.37%		7.5%	3.5%	
**H19 rs217727**						
**CC**	70%	66%	0.098	72.5%	70.1%	0.273
**CT**	22.5%	30.5%		22.5%	27.2%	
**TT**	7.5%	3.5%		5%	2.7%	

Results are presented as percent. rs, reference single-nucleotide polymorphism (SNP) ID. Abbreviations: cerebral ischemic stroke (CIS), hypertensive (HTN).

**Table 5:** Genotype and allele frequency of MIAT-rs2331291 and H19-rs217727 polymorphic sites in CIS patients and healthy controls, n (%).

Genotype Allele	CIS (n = 80)	Control (n = 40)	*p*-Value
**MIAT rs2331291**			
**CC**	42 (52.5%)	33 (82.5%)	0.02 *
**CT**	24 (30%)	7 (17.5%)	
**TT**	14 (17.5%)	0 (0%)	
**C**	108 (67.5%)	73 (91.3%)	0.0001 *
**T**	52 (32.5%)	7 (8.8%)	
**H19 rs217727**			
**CC**	57 (71.3%)	23 (57.5%)	0.22
**CT**	18 (22.5%)	15 (37.5%)	
**TT**	5 (6.3%)	2 (5%)	
**C**	132 (82.5%)	61 (76.3%)	0.25
**T**	28 (17.5%)	19 (23.8)	

Results are expressed as number and percent. rs, reference single-nucleotide polymorphism (SNP) ID. Abbreviation: cerebral ischemic stroke (CIS), * Indicates a statistical significance.

**Table 6:** Genotype and allele frequency of MIAT-rs2331291 and H19-rs217727 polymorphic sites in hypertensive and non-hypertensive CIS patients, n (%).

Genotype Allele	CIS		*p*-Value
	HTN (n = 40)	Non-HTN (n = 40)	
**MIAT rs2331291**			
**CC**	14 (35%)	28 (70%)	0.004 *
**CT**	15 (37.5%)	9 (22.5%)	
**TT**	11 (27.5%)	3 (7.5%)	
**C**	43 (53.8%)	65 (81.3%)	0.0001 *
**T**	37 (46.3%)	15 (18.7%)	
**H19 rs217727**			
**CC**	28 (70%)	29 (72.5%)	0.89
**CT**	9 (22.5%)	9 (22.5%)	
**TT**	3 (7.5%)	2 (5%)	
**C**	65 (81.3%)	67 (83.8%)	0.677
**T**	15 (18.8%)	13 (16.3%)	

Results are expressed as number and percent. rs, reference single-nucleotide polymorphism (SNP) ID. Abbreviations: cerebral ischemic stroke (CIS), hypertensive (HTN). * Indicates a statistical significance at *p* < 0.05.

**Table 7:** Relative serum expression levels of long non-coding MIAT and H19 in CIS patients.

LncRNA	Fold Change		*p*-Value
**Downregulated**			
**MIAT**	0.569 ± 0.94		0.0001 *
	**HTN (n = 40)**	**Non-HTN (n = 40)**	
**MIAT**	0.384 ± 0.55	0.754 ± 1.19	0.08
**Upregulated**			
**H19**	23.19 ± 20.87		0.0001 *
	**HTN (n = 40)**	**Non-HTN (n = 40)**	
**H19**	15.99 ± 14.5	30.38 ± 23.79	0.002 *

Data are represented as means ± standard deviation (SD). *p*-value was calculated by the Mann–Whitney U test. Abbreviations: cerebral ischemic stroke (CIS), hypertensive (HTN). * Indicates a statistical significance at *p* < 0.05.

**Table 8:** Serum expression levels of long non-coding MIAT and H19 in different genotypes of MIAT rs2331291 and H19 rs217727 in CIS patients.

Genotype Allele	CIS (n = 80)	*p*-Value
**MIAT rs2331291**		
**CC** (n = 42)	0.77 ± 1.2	0.157
**CT** (n = 24)	0.2 ± 0.17	
**TT** (n = 14)	0.56 ± 0.46	
**H19 rs217727**		
**CC** (n = 57)	21.57± 19.14	0.793
**CT** (n = 18)	28.3 ± 24.9	
**TT** (n = 5)	23.04 ± 25.9	

Data are represented as means ± standard deviation (SD). *p*-value was calculated by the Mann–Whitney U test. Abbreviations: cerebral ischemic stroke (CIS).

**Table 9:** Serum expression levels of long non-coding MIAT and H19 in different genotypes of MIAT rs2331291 and H19 rs217727 in hypertensive and non-hypertensive CIS patients.

Genotype Allele	HTN (n = 40)	*p*-Value	Non-HTN (n = 40)	*p*-Value
**MIAT rs2331291**				
**CC**	0.4 ± 0.77 (n = 14)	0.157	0.96 ± 1.37 (n = 28)	0.564
**CT**	0.19 ± 0.18 (n = 15)		0.22 ± 0.16 (n = 9)	
**TT**	0.61 ± 0.51 (n = 11)		0.39 ± 0.17 (n = 3)	
**H19 rs217727**				
**CC**	15.33 ± 11.8(n = 28)	0.128	27.58 ± 22.8 (n = 29)	0.304
**CT**	22 ± 21.4 (n = 9)		34.7 ± 27.7 (n = 9)	
**TT**	4.11 ± 0.57 (n = 3)		51.4 ± 0.0 (n = 2)	

Data are represented as means ± standard deviation (SD). *p*-value was calculated by the Mann–Whitney U test. Abbreviations: cerebral ischemic stroke (CIS), hypertensive (HTN).

**Table 10:** Serum expression levels of long non-coding H19 in different genotypes of H19-rs217727 promoter methylation in CIS patients and healthy controls.

	CIS (n = 80)		Control (n = 40)
**Expression Level**	53.79 ± 5.45		45.4 ± 0.6
***p*-Value**	0.0001 *		
**Genotype Allele**			
**CC**	53.89 ± 5.3 (n = 57)		45.5 ± 0.63 (n = 23)
**CT**	53.9 ± 6 (n = 18)		45.1 ± 0.6 (n = 15)
**TT**	51.9 ± 5.2 (n = 5)		45.2 ± 0.8 (n = 2)
***p*-Value**	0.817		0.261
**Genotype Allele**	**HTN (n = 40)**	**Non-HTN (n = 40)**	
**CC**	53.5 ± 5.1 (n = 28)	54.26 ± 5.5 (n = 29)	
**CT**	53.1 ± 6 (n = 9)	54.8 ± 6.3 (n = 9)	-------
**TT**	49.8 ± 3.2 (n = 3)	54.9 ± 7.6 (n = 2)	-------
***p*-Value**	0.598	0.854	-------

Data are represented as means ± standard deviation (SD). *p*-value was calculated by the Mann–Whitney U test. Abbreviations: cerebral ischemic stroke (CIS), hypertensive (HTN). * Indicates a statistical significance at *p* < 0.05.

**Table 11:** Correlation of serum lncRNA-MIAT and H19 levels in hypertensive and non-hypertensive CIS patients.

	CIS			
	HTN (n = 40)		Non-HTN (n = 40)	
**r**	0.284		0.259	
***p*-value**	0.07		0.107	
	**D.M** **(n = 20)**	**Non-D.M** **(n = 20)**	**D.M** **(n = 18)**	**Non-D.M** **(n = 22)**
**r**	0.045	0.646	0.017	0.422
***p*-value**	0.85	0.002 *	0.945	0.064

r, correlation coefficient. Abbreviations: cerebral ischemic stroke (CIS), hypertensive (HTN), diabetic mellitus (D.M), * Indicates a statistical significance at *p* < 0.05.

## Data Availability

All data generated or analyzed during this study are included in this published article and its Supplementary Information files.

## Supplementary Materials

The following supporting information can be downloaded at: https://www.mdpi.com/article/10.3390/ijms25020842/s1.
