# Supplementary material for: Expression, Functional Polymorphism, and Diagnostic Values of MIAT rs2331291 and H19 rs217727 Long Non-Coding RNAs in Cerebral Ischemic Stroke Egyptian Patients
Source: Int J Mol Sci. 2024 Jan 10;25(2):842. doi: 10.3390/ijms25020842 (PMC10815378; doi:10.3390/ijms25020842)
Supplement: Supplementary file 1 [file ijms-25-00842-s001.zip › Table S1.pdf]

Table S1. Hardy-Weinberg equilibrium for MIAT-rs2331291 and H19-rs217727 in diabetes mellitus and non- diabetes mellitus hypertensive CIS patients

Genotype allele	HTN					
	D.M (n= 20)			Non-D.M (n= 20)		
**MIAT rs2331291**	Observed frequency	Expected frequency	p-value	Observed frequency	Expected frequency	p-value
**CC**	40%	27.5%	0.026*	30%	30.3%	0.964
**CT**	25%	50%		50%	49.5%	
**TT**	35%	22.5%		20%	20.2%	
**H19 rs217727**						
**CC**	70%	64%	0.094	70%	68%	0.548
**CT**	20%	32%		25%	28.9%	
**TT**	10%	4%		5%	3.1%	

Results are presented as percent. *rs,* reference single-nucleotide polymorphism (SNP) ID, Abbreviations: cerebral ischemic stroke (CIS), hypertensive (HTN), diabetic mellitus (D.M), * Indicates a statistical significance at $p < 0.05$.
