# Supplementary material for: Expression, Functional Polymorphism, and Diagnostic Values of MIAT rs2331291 and H19 rs217727 Long Non-Coding RNAs in Cerebral Ischemic Stroke Egyptian Patients
Source: Int J Mol Sci. 2024 Jan 10;25(2):842. doi: 10.3390/ijms25020842 (PMC10815378; doi:10.3390/ijms25020842)
Supplement: Supplementary file 1 [file ijms-25-00842-s001.zip › Table S2.pdf]

Table S2. Hardy-Weinberg equilibrium for MIAT-rs2331291 and H19-rs217727 in diabetes mellitus and non- diabetes mellitus non-hypertensive CIS patients

Genotype allele	Non- HTN					
	D.M (n=20)			Non-D.M (n=20)		
**MIAT rs2331291**	Observed frequency	Expected frequency	p-value	Observed frequency	Expected frequency	p-value
**CC**	61.2%	56.3%	0.271	77.3%	74.6%	0.285
**CT**	27.8%	37.5%		18.2%	23.5%	
**TT**	11%	6.2%		4.5%	1.9%	
**H19 rs217727**						
**CC**	72.2%	69.4%	0.396	72.5%	70.7%	0.48
**CT**	22.2%	27.8%		22.7%	26.8%	
**TT**	5.6%	2.8%		4.6%	2.5%	

Results are presented as percent. *rs,* reference single-nucleotide polymorphism (SNP) ID, Abbreviations: cerebral ischemic stroke (CIS), hypertensive (HTN), diabetic mellitus (D.M).
