# Supplementary material for: Expression, Functional Polymorphism, and Diagnostic Values of MIAT rs2331291 and H19 rs217727 Long Non-Coding RNAs in Cerebral Ischemic Stroke Egyptian Patients
Source: Int J Mol Sci. 2024 Jan 10;25(2):842. doi: 10.3390/ijms25020842 (PMC10815378; doi:10.3390/ijms25020842)
Supplement: Supplementary file 1 [file ijms-25-00842-s001.zip › Table S3.pdf]

Table S3. Genotype and allele frequency of MIAT and H19 polymorphic sites in hypertensive and non-hypertensive CIS patients, n (%)

Genotype allele	HTN (n=40)			Non- HTN (n=40)		
	D.M (n=20)	Non- D.M (n=20)	p-value	D.M (n=20)	Non- D.M (n=20)	p-value
**MIAT rs2331291**						
**CC**	8 (40%)	6 (30%)	0.25	11(61.2%)	17 (77.3%)	0.511
**CT**	5 (25%)	10 (50%)		5 (27.8%)	4 (18.2%)	
**TT**	7 (35%)	4 (20%)		2 (11%)	1 (4.5%)	
**C**	21 (52.5%)	22 (55%)	0.823	27 (75%)	38 (86.4%)	0.195
**T**	19 (47.5%)	18 (45%)		9 (25%)	6 (13.6%)	
**H19 rs217727**						
**CC**	14 (70%)	14 (70%)	0.8	13 (72.2%)	16 (72.7%)	0.989
**CT**	4 (20%)	5 (25%)		4 (22.2%)	5 (22.7%)	
**TT**	2 (10%)	1 (5%)		1(5.6%)	1 (4.6%)	
**C**	32 (80%)	33 (82.5%)	0.775	30 (83.3%)	37 (84.1%)	0.927
**T**	8 (20%)	7 (17.5%)		6 (16.7%)	7 (15.9)	

Results are expressed as number and percent. rs, reference single nucleotide polymorphism (SNP) ID, Abbreviations: cerebral ischemic stroke (CIS), hypertensive (HTN).
